# PRPF8 increases the aggressiveness of hepatocellular carcinoma by regulating FAK/AKT pathway via fibronectin 1 splicing

**DOI:** 10.1038/s12276-022-00917-7

**Published:** 2023-01-06

**Authors:** Juan L. López-Cánovas, Natalia Hermán-Sánchez, Mercedes del Rio-Moreno, Antonio C. Fuentes-Fayos, Araceli Lara-López, Marina E. Sánchez-Frias, Víctor Amado, Rubén Ciria, Javier Briceño, Manuel de la Mata, Justo P. Castaño, Manuel Rodriguez-Perálvarez, Raúl M. Luque, Manuel D. Gahete

**Affiliations:** 1 Maimónides Institute of Biomedical Research of Córdoba (IMIBIC), 14004 Córdoba, Spain; 2 Department of Cell Biology, Physiology and Immunology, University of Córdoba, 14004 Córdoba, Spain; 3 Reina Sofía University Hospital, 14004 Córdoba, Spain; 4 CIBER Pathophysiology of Obesity and Nutrition (CIBERobn), 14004 Córdoba, Spain; 5 Department of Hepatology and Liver Transplantation, Reina Sofía University Hospital, 14004 Córdoba, Spain; 6 CIBER Hepatic and Digestive Diseases (CIBERehd), 14004 Córdoba, Spain; 7 Unit of Hepatobiliary Surgery and Liver Transplantation, University Hospital Reina Sofia, 14004 Cordoba, Spain

**Keywords:** Mechanisms of disease, Liver cancer, Transcriptomics

## Abstract

Hepatocellular carcinoma (HCC) pathogenesis is associated with alterations in splicing machinery components (spliceosome and splicing factors) and aberrant expression of oncogenic splice variants. We aimed to analyze the expression and potential role of the spliceosome component PRPF8 (pre-mRNA processing factor 8) in HCC. PRPF8 expression (mRNA/protein) was analyzed in a retrospective cohort of HCC patients (*n* = 172 HCC and nontumor tissues) and validated in two in silico cohorts (TCGA and CPTAC). *PRPF8* expression was silenced in liver cancer cell lines and in xenograft tumors to understand the functional and mechanistic consequences. In silico RNAseq and CLIPseq data were also analyzed. Our results indicate that PRPF8 is overexpressed in HCC and associated with increased tumor aggressiveness (patient survival, etc.), expression of HCC-related splice variants, and modulation of critical genes implicated in cancer-related pathways. *PRPF8* silencing ameliorated aggressiveness in vitro and decreased tumor growth in vivo. Analysis of in silico CLIPseq data in HepG2 cells demonstrated that PRPF8 binds preferentially to exons of protein-coding genes, and RNAseq analysis showed that *PRPF8* silencing alters splicing events in multiple genes. Integrated and in vitro analyses revealed that *PRPF8* silencing modulates fibronectin (FN1) splicing, promoting the exclusion of exon 40.2, which is paramount for binding to integrins. Consistent with this finding, *PRPF8* silencing reduced FAK/AKT phosphorylation and blunted stress fiber formation. Indeed, HepG2 and Hep3B cells exhibited a lower invasive capacity in membranes treated with conditioned medium from *PRPF8*-silenced cells compared to medium from scramble-treated cells. This study demonstrates that PRPF8 is overexpressed and associated with aggressiveness in HCC and plays important roles in hepatocarcinogenesis by altering FN1 splicing, FAK/AKT activation and stress fiber formation.

## Liver cancer: Targeting splicing protein could slow tumour growth

The overexpression of a splicing-related protein is directly linked to tumour aggressiveness in liver cancer, suggesting a potential treatment target. mRNA splicing is a celullar process that enables a single gene to generate multiple differerent proteins. However, in cancers aberrant gene splicing can facilitate disease progression and tumour growth. In experiments on human samples, cell lines and animal models, Manuel D. Gahete at the Maimónides Institute of Biomedical Research of Córdoba, Spain, and co-workers investigated the role of PRPF8, a key component of the protein complex that drives the splicing process in liver cancer. PRPF8 overexpression directly influences cancer aggressiveness and alters cancer-related splicing variants, particularly fibronectin 1, a glycoprotein found in the extracellular matrix. Silencing the *PRPF8* gene decreased tumour growth and aggressiveness, indicating such an approach could prove useful in treating liver cancer.

## Introduction

Hepatocellular carcinoma (HCC) is the most prevalent type of primary liver cancer and the fourth most common cancer worldwide^1^. The majority of HCC cases are associated with chronic liver diseases due to alcohol consumption, chronic viral hepatitis or metabolic syndrome, among other etiologies^1^. Since the 1990s, the incidence of HCC has increased dramatically, and HCC-related mortality is increasing faster than that of other cancer types^1^. The elevated death rates observed for HCC could be associated with its late diagnosis despite routine screening strategies with liver ultrasound every 6 months and because the available therapies have a limited impact on overall survival^2^. Therefore, a better understanding of hepatocellular carcinogenesis could help to identify new diagnostic, prognostic and therapeutic targets.

A common hallmark of cancer is the alteration of important elements regulating cell physiology, especially the presence or aberrant expression of splice variants, which could be associated with development, progression and drug resistance in different types of cancer^3^. In fact, splice variants of important genes such as *CDCC50*, *KLF6*, and *FN1*^4,5^ are involved in liver carcinogenesis, thus suggesting that an altered splicing process could play an essential role in the development and progression of HCC^6^.

The splicing process is controlled by the spliceosome, a macromolecular ribonucleoprotein complex that cooperates with hundreds of splicing factors to catalyze this process^7^. This splicing machinery is essential for appropriate modulation of gene expression, and its dysregulation is associated with oncogenic progression, including in HCC^8^, and with the generation of an aberrant landscape of alternative splice variants^9^. In this context, the splicing factor PRPF8 (pre-mRNA processing factor 8) is a key protein in the catalytic nucleus of the spliceosome, which participates in the second step of the splicing process. In *Drosophila*, loss of PRPF8 decreases cell proliferation, increases cell death and modulates cell differentiation and polarity, and PRPF8-mediated hyperplastic growth is induced by different oncogenes^10^. Indeed, PRPF8 seems to be crucial for appropriate constitutive and alternative mRNA splicing^11^. PRPF8 mutations have been associated with severe forms of retinitis pigmentosa as well as with the initiation of various types of myeloid neoplasms and with decreased survival in patients with leukemia^12^. In solid tumors, silencing of *PRPF8* was found to result in cancer subtype-specific implications in breast cancer cell lines^13^, while in prostate cancer, PRPF8 is involved in androgen receptor splicing^14^. In addition, recent evidence suggests that PRPF8 may also be overexpressed in HCC and be associated with the tumorigenic potential; however, these conclusions were based on a single HCC cohort and experiments with a single HCC cell line^15^. Based on this information, and using several in vitro approaches, animal models, and human samples, we aimed to explore the putative dysregulation, association with clinical parameters and functional role of PRPF8 in an ample number of HCC cohorts and cell lines; the implication of PRPF8 in the control of the splicing process in HCC; and the potential utility of its genetic modulation in hepatocarcinogenesis.

## Material and methods

### Patients and samples

A retrospective cohort of patients with HCC who underwent surgical resection or liver transplantation was included. Formalin-fixed, paraffin-embedded (FFPE) samples containing paired HCC and nontumor adjacent tissues from *n* = 86 patients were included. All samples were obtained from the Andalusian Biobank (Cordoba Node) and were histologically evaluated and diagnosed by two independent pathologists. Clinical data of the patients are shown in Table 1. The study protocol was approved by the Reina Sofia University Hospital Ethics Committee and was conducted according to institutional and Good Clinical Practice guidelines (protocol number PI17/02287) and in compliance with the Declaration of Helsinki. Informed consent was obtained from all patients or their relatives.

**Table 1:** Demographic and clinical parameters of HCC patients included in the retrospective cohort.

Patients [*n*]	86
Age, y [median (IQR)]	60.6 (64–67)
Etiology [*n* (%)]	
− HCV	30 (36.1)
− Alcohol	21 (25.3)
− HBV	11 (13.3)
− Other	5 (6)
− HCV + Alcohol	9 (10.8)
− HBV + Alcohol	1 (1.2)
− HCV + other	3 (3.6)
− Alcohol + other	0(-)
Histological differentiation [*n* (%)]	
− Well differentiated	30 (35.3)
− Moderately differentiated	50 (58.8)
− Poorly differentiated	5 (5.9)
Portal hypertension [*n* (%)]	44 (51.2)
Microvascular invasion [*n* (%)]	33 (39.8)
Treated before surgery [*n* (%)]	23 (26.4)
Recurrence [*n* (%)]	39 (47)
Death [*n* (%)]	50 (61)

GEPIA2, an enhanced web server for large-scale expression profiling and interactive analysis, was used to analyze the expression level (RNAseq data) of *PRPF8* in tumor (*n* = 369) and normal (*n* = 50) tissues and the survival of HCC patients from the TCGA cohort. The Cancer Institute Clinical Proteomic Tumor Analysis Consortium (CPTAC) portal was used to download PRPF8 protein expression data from proteomic studies and to analyze the differences in PRPF8 levels between paired tumor and adjacent liver tissues from 165 patients with HBV-related HCC (Zhou Cohort). In this cohort, tumor differentiation was graded according to the Edmondson system^16^.

### Cell lines and treatments

The liver cancer cell lines HepG2, Hep3B and SNU-387 (HB-8065) were used (ATCC, Manassas, USA). HepG2 and Hep3B cells were cultured in minimum essential medium (Thermo Fisher, Madrid, Spain) supplemented with 10% fetal bovine serum (FBS; Sigma‒Aldrich, Madrid, Spain), 0.2% antibiotic-antifungal (Gentamicin/amphotericin-B, Thermo Fisher) and 0.5% sodium pyruvate^8^. SNU-387 cells were cultured in Roswell Park Memorial Institute 1640 (RPMI-1640; Thermo Fisher) medium supplemented with 10% FBS, 0.2% antibiotic-antifungal and 0.5% glutamine (Thermo Fisher)^8^. Cells were maintained at 37 °C and 5% CO_2_ under sterile conditions, periodically validated by short tandem repeat analysis (GenePrint 10 System, Promega, Barcelona, Spain) and tested for mycoplasma contamination^8,17,18^. Positive and/or negative controls [IGF-1 (10^-6^ M) and paclitaxel (10^-7^ M), respectively] were used.

### *PRPF8* silencing by specific siRNAs and *PRPF8* overexpression

A small interfering RNA against *PRPF8* (s20796, Thermo Fisher) and a negative control siRNA (scramble; Thermo Fisher) were used. For transfection, 120,000 SNU-387 and 150,000 Hep3B or HepG2 cells were seeded in 6-well plates^8^. The medium was replaced with antibiotic/antimycotic-free medium, and the cells were transfected with 50 nM *PRPF8* siRNA (*siPRPF8*) using Lipofectamine RNAiMAX reagent (Thermo Fisher). After 48 h, the medium was collected, and the cells were detached and seeded for extraction of RNA and protein and for functional assays. For PRPF8 overexpression, specific plasmids (pcDNA3.1+) were used. For transfection, 150,000 Hep3B cells were seeded in 6-well plates. The empty pCDNA3.1+(mock transfected) vector was used as a negative control. After 24 h, the cells were detached and seeded for RNA and protein extraction.

### Confocal microscopy

Transfected HepG2 and Hep3B cells grown on coverslips were fixed with 4% paraformaldehyde and incubated with Alexa Fluor488-phalloidin (A12379, Thermo Fisher) for 15 minutes. The samples were counterstained with DAPI (Sigma‐Aldrich) and mounted with mounting medium (Dako)^19^. Cell preparations were visualized under an LSM710 confocal laser scanning microscope (Carl Zeiss, Jena, Germany; Microscopy facility, IMIBIC). An overlapping pixel map of signals in the fluorescence channels was generated with ImageJ.

### In vitro, in silico, PCR and western blot analyses

Measurements of cell proliferation, migration and formation of clones and tumorspheres were performed as previously described^8^. The procedures used for RNA isolation and reverse transcription, RNA expression analysis by microfluidic qPCR and conventional qPCR, semiquantitative PCR and western blotting have been previously reported^8,17,18,20–22^. More details about these approaches and about the RNAseq and CLIPseq analyses are provided in the Supplementary Material and Methods and Supplementary Table 1.

### Invasion assays

Cell invasion was evaluated by a modified Boyden chamber method using a 48-well chemotaxis chamber (NeuroProbe, Gaithersburg, MD). Briefly, 25,000 HepG2 and 20,000 Hep3B cells per well were seeded onto an 8 µM pore PDVF membrane (NeuroProbe) previously treated with collagen type IV (BD Biosciences, San Jose, CA) and conditioned medium from scramble- and *siPRPF8*-treated cells. The lower well was filled with FBS-free medium (negative control) or medium containing 10% FBS, with two replicates per condition^23^. After 24 h, the non-migrated cells were removed, and the migrated cells were fixed with crystal violet solution (6% glutaraldehyde, 0.5% crystal violet). Images of random fields were acquired and analyzed with ImageJ. The experiments were performed in triplicate.

### Xenograft model

Experiments with xenografted nude mice were carried out according to the European Regulations for Animal Care with the approval of the university research ethics committees. Eight-week-old male Foxn1nu/nu mice (Janvier Labs, Le Genest-Saint-Isle, France) were subcutaneously grafted in both flanks (*n* = 6 mice) with 5 × 10^6^ Hep3B cells in 50 µl of basement membrane extract (Trevigen, Gaithersburg, MD)^8^. Tumor growth and mouse weights were monitored twice per week. Three weeks post grafting, when the tumors were visible, each tumor was covered with 10 µM scramble or si*PRPF8* diluted in 50 µl of AteloGene (Koken, Tokyo, Japan) to induce silencing of *PRPF8*, and tumor growth was monitored^24^. After the mice were euthanized, each tumor was harvested, and different pieces were snap frozen or fixed with formalin. Tumor H&E staining was analyzed by expert pathologists.

### IHC analysis

Immunohistochemical (IHC) analysis of PRPF8 was performed on a representative set of FFPE samples of paired nontumor adjacent and HCC tissue (*n* = 14). A monoclonal anti-PRPF8 antibody (ab79237, Abcam, Cambridge, UK) diluted 1:250 was used^25^. Two independent pathologists performed histopathologic analysis of tumors following a blinded protocol. In this analysis, +, ++, and + ++ indicate a low, moderate, and high staining intensity, respectively.

### Statistical analysis

Data are expressed as the means ± standard errors of the mean (SEM), as fold changes (log 2 transformed) or as relative levels compared with the corresponding controls (set at 100%). Data were evaluated for heterogeneity of variance using the Kolmogorov–Smirnov test, and parametric (Student’s t) or nonparametric (Mann‒Whitney U) tests were implemented as appropriate. Spearman or Pearson bivariate correlation analysis was performed for quantitative variables according to the normality of the data. The significance of relationships between stratified mRNA expression level and patient survival was determined using Kaplan‒Meier analysis and log-rank-p values (groups were selected based on the cutoff points determined by the median expression level of the gene of interest). *P*-values lower than 0.05 were considered statistically significant. All statistical analyses were performed using GraphPad Prism 6.0 software (La Jolla, CA, USA).

## Results

### *PRPF8* was overexpressed in HCC, correlated with the expression of oncogenic splice variants and associated with overall survival

**Fig. 1:**
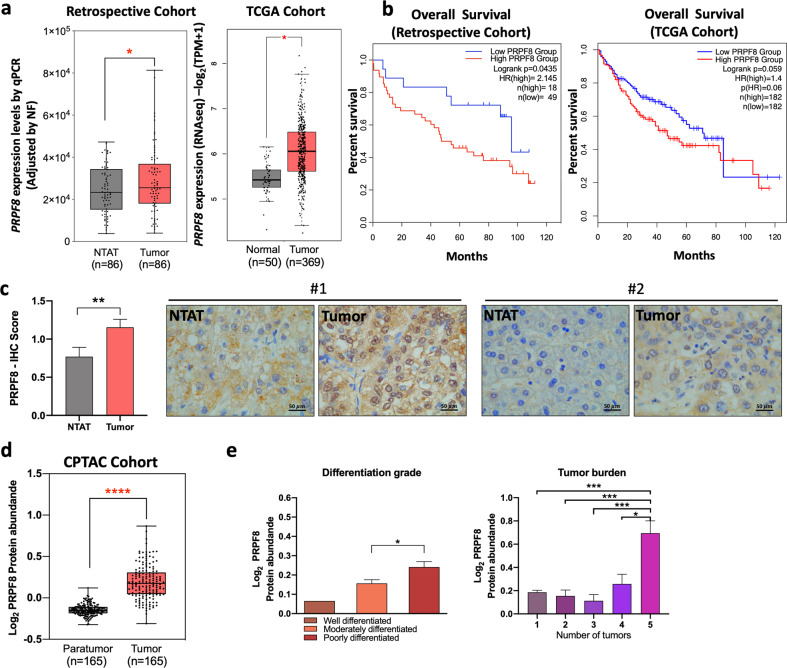
PRPF8 is overexpressed in HCC and is associated with clinical aggressiveness and poor survival in HCC patients. **a** Expression level of PRPF8 in HCC [retrospective cohort (determined by qPCR): *n* = 86 patients and TCGA cohort (obtained from RNAseq data): *n* = 419 samples]. **b** Overall survival in the retrospective and TCGA cohorts, categorized into the high and low PRPF8 expression groups based on the median PRPF8 expression level, and analyzed by determining the log-rank-*p*-value. **c** Immunohistochemical (IHC) score of PRPF8 in NTAT and tumor samples (*n* = 14 patients from the retrospective cohort). Representative images (60X) from two patients are shown. **d** Protein level of PRPF8 in paratumor and tumor tissues from CPTAC data. **e** Correlations between the PRPF8 protein level and clinical aggressiveness in the CPTAC cohort. The data are presented as the means ± SEMs. The asterisks (*p < 0,05; **p < 0,01; ***p < 0,001; ****p < 0,0001) indicate statistically significant differences. NTAT Nontumor adjacent tissue, HR Hazard ratio.

The *PRPF8* mRNA level, as determined by qPCR, was significantly higher in HCC samples than in nontumor adjacent tissues (NTATs) in the retrospective cohort (Fig. 1a), and this pattern was corroborated in silico in the TGCA cohort (RNAseq data) (Fig. 1a). The *PRPF8* mRNA level was associated with overall survival, since patients with higher *PRPF8* expression levels exhibited lower overall survival rates in the retrospective cohort (HR = 2.145, *p* = 0.0435), and the same trend was observed in the TCGA cohort (HR = 1.4, *p* = 0.06) (Fig. 1b). The results obtained in the retrospective cohort also indicated that the expression of PRPF8 is not dependent on the HCC etiology (Supplementary Fig. 1a). Interestingly, in the HCC samples from the retrospective cohort, the *PRPF8* expression level was directly correlated with the expression of important oncogenic HCC-related splice variants, such as *CCDC50S* and *KLF6SV1* (*R* > 0.303; *p* < 0.001 in both cases; Supplementary Fig. 1b). In addition, the PRPF8 protein level, as assessed by IHC staining, was significantly higher in the nuclei of HCC samples compared to NTATs in the retrospective cohort (Fig. 1c). Consistent with this finding, in silico proteomic data available via the CPTAC portal indicated that the PRPF8 protein level was higher in HCC samples than in paratumor tissues (Fig. 1d). In this cohort, the PRPF8 protein level was correlated with the AFP level (Supplementary Fig. 1c), differentiation grade, and the number of tumors (Fig. 1e).

### Modulation of *PRPF8* altered the expression of multiple genes involved in key cancer-related pathways

**Fig. 2:**
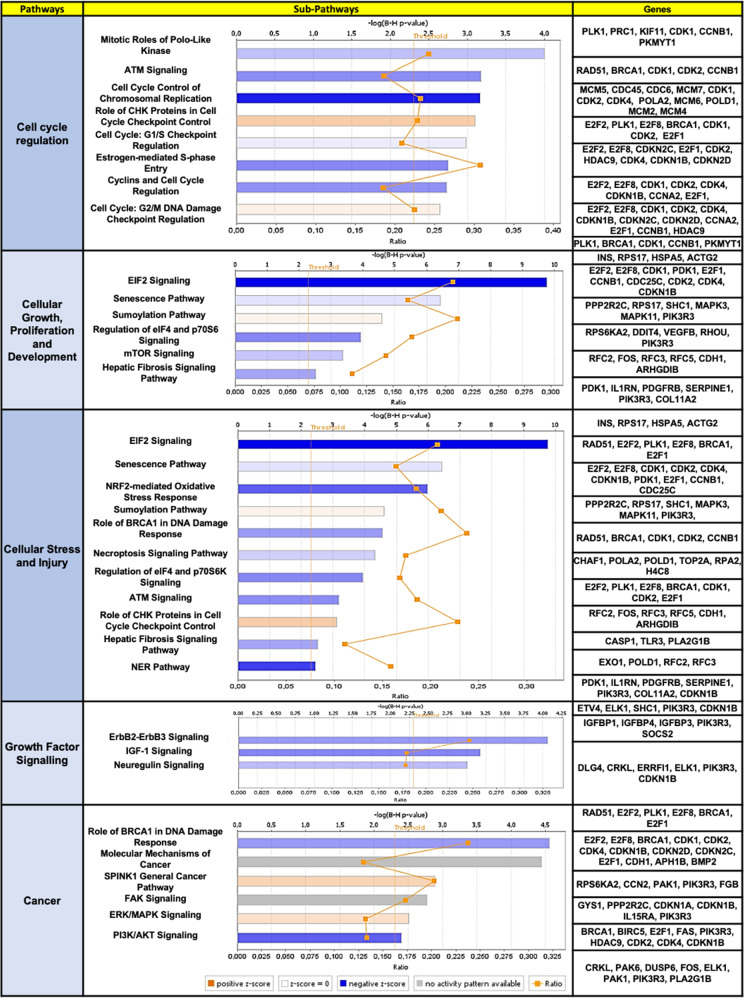
PRPF8 silencing alters the expression of several genes involved in key cancer-related pathways. RNAseq data from PRPF8-silenced HepG2 cells vs. control cells were processed and analyzed by Ingenuity Pathway Analysis. Key pathways and subpathways altered in response to PRPF8 silencing are shown. The bars indicate the -log(B-H p values), and the significance threshold was calculated as <0.05. The most representative altered genes in each subpathway are shown.

An exhaustive analysis of in silico RNAseq data from *PRPF8*-silenced HepG2 cells revealed a total of 3170 differentially expressed genes (DEGs) between non-silenced and silenced HepG2 cells (*p*-value < 0.01 and shown in the volcano plot in Supplementary Fig. 2a). Analysis of these DEGs using IPA revealed significant alterations in key cancer-related pathways, such as cell cycle, cellular growth and cellular stress, showing the general inactivation of oncogenic pathways and hyperactivation of tumor suppressor pathways (Fig. 2).

### *PRPF8* silencing reduced aggressive features in liver cancer cell lines

**Fig. 3:**
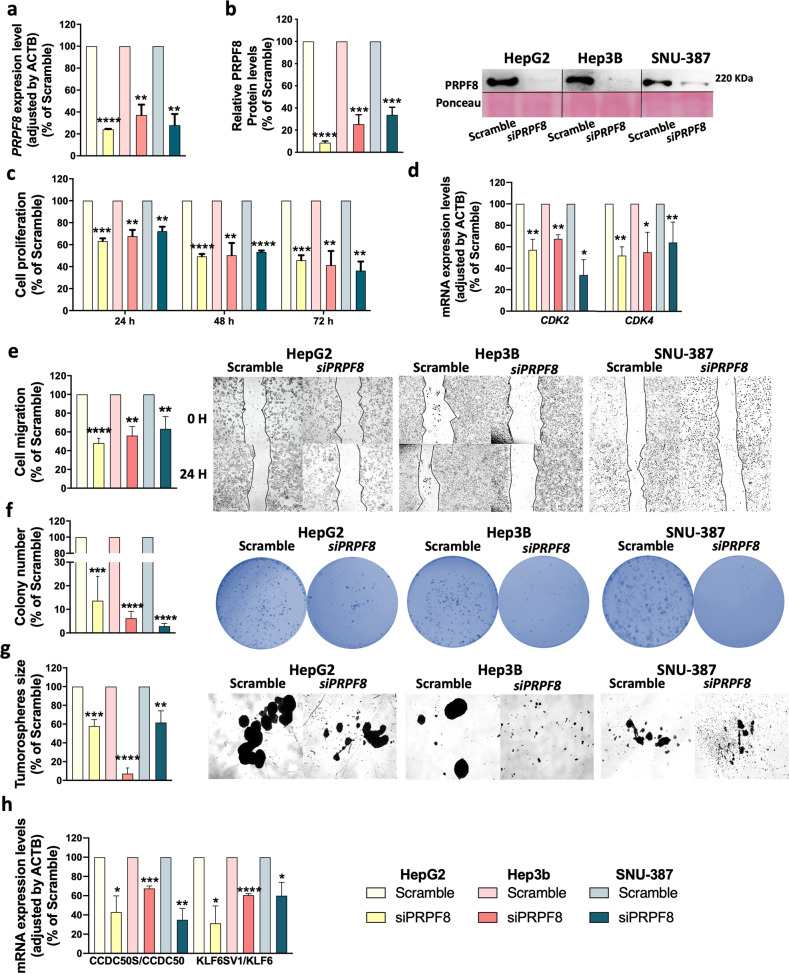
PRPF8 modulation by a specific siRNA decreases the aggressive features of liver cancer cell lines. Validation of siRNA-mediated *PRPF8* silencing at the mRNA (**a**) and protein (**b**) levels. **c** Proliferation of *PRPF8*-silenced cells compared to scramble-treated cells. **d** mRNA expression levels of key cell cycle-related genes (CDK2 and CDK4) in *PRPF8*-silenced vs. scramble-treated cells. **e** Migration of *PRPF8*-silenced cells compared to scramble-treated cells. Representative images at 0 h and after 24 h are shown. **f** Number of colonies formed in *PRPF8*-silenced vs. scramble-treated cells. Representative images of colonies formed after 10 days are shown. **g** Mean size of tumorspheres formed from *PRPF8*-silenced vs. scramble-treated cells. Representative images of tumorspheres formed after 10 days are shown. **h** Ratios of mRNA expression levels of key oncogenic splice variants and the corresponding full-length transcripts (*CCDC50S/CCDC50* and *KLF6SV1/KLF6*) in *PRPF8*-silenced vs. scramble-treated cells. The data are presented as the mean ± SEM of *n* = 3–5 independent experiments. The asterisks (*p < 0,05, **p < 0,01; ***p < 0,001, ****p < 0,0001) indicate statistically significant differences.

To analyze the implication of PRPF8 in HCC behavior, a specific siRNA (*siPRPF8*) was used to reduce the expression level of PRPF8, which was confirmed at the mRNA and protein levels in all cell lines (Fig. 3a, b). Silencing *PRPF8* significantly reduced cell proliferation at 24–72 h in all cell lines tested (Fig. 3c). These results were in line with the reductions in the expression of key cell cycle regulators, such as *CDK2* and *CDK4*, in vitro (Fig. 3d). In addition, the wound healing assay demonstrated that *siPRPF8* significantly reduced the migratory capacity of all cell lines (Fig. 3e). Moreover, the clonogenic assay demonstrated that the number of colonies formed was significantly lower (Fig. 3f) and the mean size of the tumorspheres was markedly reduced in all cell lines in response to *PRPF8* silencing (Fig. 3g). Interestingly, *PRPF8* silencing induced selective reductions in the expression of key oncogenic splice variants found to be correlated with *PRPF8* expression in human HCC samples. Indeed, in vitro silencing of *PRPF8* reduced the *CCDC50S/CCDC50* and *KLF6SV1/KLF6* ratios in the three HCC cell lines used, indicating lower expression of the oncogenic splice variants (Fig. 3h).

### In vivo *PRPF8* silencing reduced the growth of xenografted liver cancer cell lines in nude mice

**Fig. 4:**
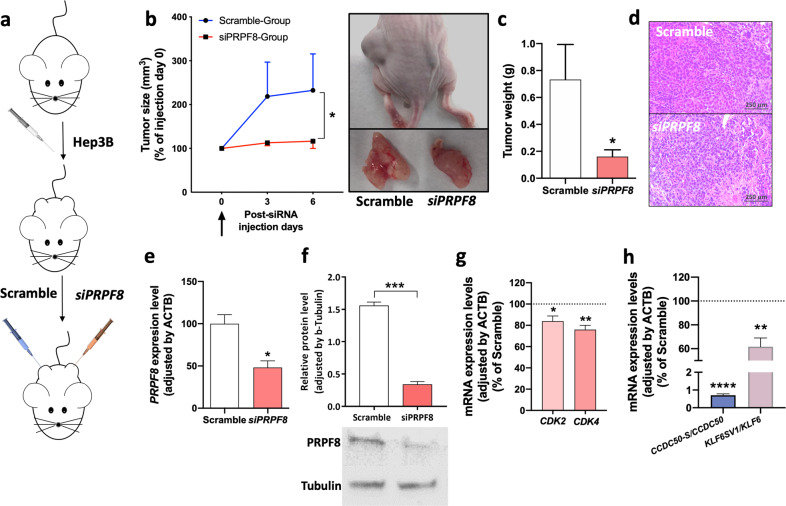
PRPF8 silencing decreases HCC cell growth in vivo. **a** Diagram showing the in vivo experimental design. At the third week post grafting, each tumor was transfected with scramble siRNA or siPRPF8 (*n* = 6 mice). **b** The growth rate of tumors was determined throughout the first 6 days after transfection. Representative images of scramble- and siPRPF8-treated tumors are shown. **c** The final tumor weight was calculated after sacrifice. **d** Tumor tissue hematoxylin and eosin (H&E) staining. **e** Validation of the siRNA-mediated reduction in *PRPF8* mRNA expression. **f** Validation of the siRNA-mediated reduction in *PRPF8* protein expression. **g** mRNA expression levels of key cell cycle-related genes (CDK2 and CDK4) in *PRPF8*-silenced vs. scramble-treated cells. **h** Ratios of mRNA expression levels between key oncogenic splice variants and the corresponding full-length transcripts (*CCDC50S/CCDC50* and *KLF6SV1/KLF6*) in *PRPF8*-silenced vs. scramble-treated xenografts. The asterisks (*p < 0,05; **p < 0,01) indicate statistically significant differences.

The effect of *PRPF8* silencing on HCC growth in vivo was evaluated in Hep3B-induced xenografts (Fig. 4a). Specifically, in vivo silencing of *PRPF8* by *siPRPF8* in established s.c. tumors significantly reduced tumor growth (Fig. 4b) and the final tumor weight (Fig. 4c) compared to those of scramble-treated tumors. However, the histology of control and *PRPF8*-silenced tumors did not show evident differences (Fig. 4d). The effectiveness of *PRPF8* silencing was validated at the mRNA (Fig. 4e) and protein (Fig. 4f) levels. In addition, *PRPF8* silencing was associated with reductions in *CDK2* and *CDK4* expression in Hep3B xenografts (Fig. 4g), accompanied by reductions in the expression of key oncogenic splice variants found to be correlated with *PRPF8* expression in human HCC samples, such as *CCDC50S* and *KLF6SV1* (Fig. 4h).

### PRPF8 interacts with exonic regions of key cancer-related genes, modifying their splicing patterns

**Fig. 5:**
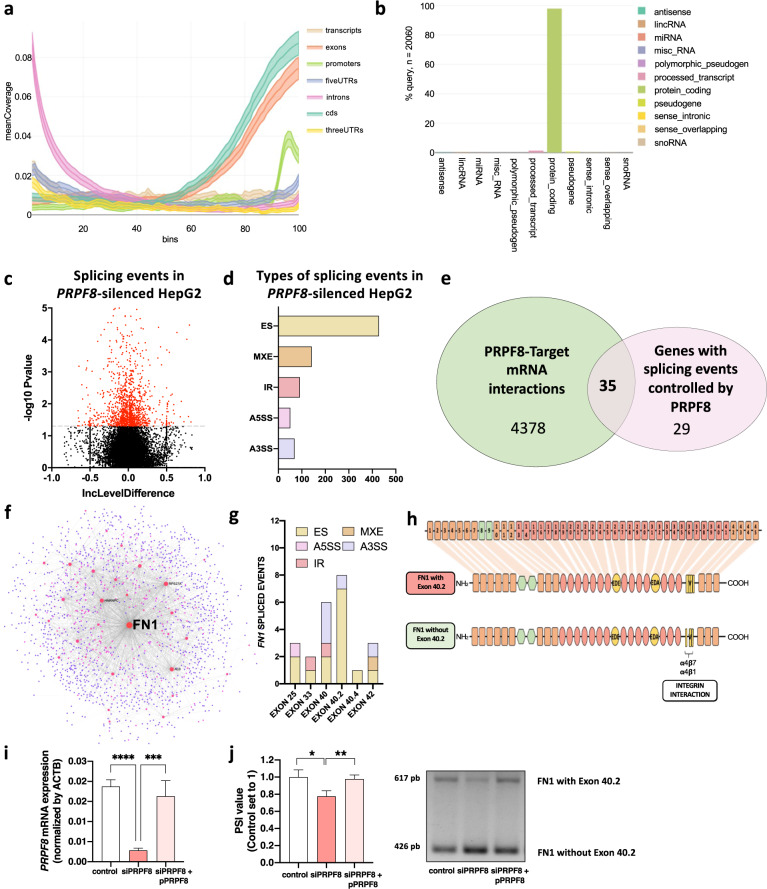
PRPF8 binds to exonic regions in protein-coding transcripts to modulate splicing. Coverage profile of the preferred binding location of PRPF8 (**a**) and transcript features (**b**) determined from in silico analysis of eCLIP data for PRPF8 in HepG2 cells. **c** Volcano plot of splicing events altered by silencing of *PRPF8* in HepG2 cells. Data were obtained from ENCODE and analyzed with *rmats*. **d** Number of events of each type of splicing induced by PRPF8 silencing in HepG2 cells. **e** Venn diagram of PRPF8-target mRNA interactions (CLIPseq data) and splicing events induced by *PRPF8* silencing (RNAseq data) showing that 35 genes whose mRNA is targeted by PRPF8 exhibit altered splicing events in response to PRPF8 silencing. **f** Protein‒protein interaction analysis^26^ identified fibronectin 1 (FN1) as a hub gene. **g** Splicing events found in the FN1 gene in response to *PRPF8* silencing, among which exon 40.2 skipping was the most common altered event. RNAseq data from HepG2 cells were obtained and analyzed. **h** Diagram showing the skipping of FN1 exon 40.2, which encodes an integrin interaction region. **i** Validation of PRPF8 expression levels by qPCR in silencing (siPRPF8) and rescue (siPRPF8 + pPRPF8) experiments in Hep3B cells, with comparison to those in mock transfected cells. **j** Evaluation of the FN1 exon 40.2 inclusion PSI value by PCR in response to PRPF8 silencing and PRPF8 silencing/overexpression compared with that in scramble-treated/mock Hep3B cells. PSI (percent spliced in) indicates the inclusion of an exon. ES Exon skipping, MXE Mutually exclusive exons, A5SS Alternative 5´ splice site, A3SS Alternative 3´ splice site, IR Intron retention. The asterisks (*p < 0,05; **p < 0,01, ***p < 0,001, ****p < 0,0001) indicate statistically significant differences.

Analysis of available in silico eCLIP data in HepG2 cells in The Encyclopedia of RNA Interactomes (ENCORI) with RCAS (a R/Bioconductor package) showed that PRPF8 is capable of binding to a large number of nascent mRNAs (4410 interactions). PRPF8 most preferentially binds to exonic sequences and, to a lesser extent, to 3´ UTRs (Fig. 5a and Supplementary Fig. 2b–d). These regions mostly correspond to exons of protein-coding genes (Fig. 5b). Consistent with this finding, a splicing event-focused analysis of the RNAseq data available for HepG2 cells revealed that modulation of *PRPF8* expression induced changes in a total of 782 splicing events (*p*-value < 0.05) in *PRPF8*-silenced HepG2 cells (Fig. 5c), most of which were exon skipping (ES) events (Fig. 5d). Combined analysis of those genes whose mRNA can be bound by *PRPF8* (4410 genes) with those genes whose splicing events are modulated by *PRPF8* silencing (782 splicing events in 57 genes) revealed the existence of 35 genes whose nascent mRNA physically interacts with PRPF8 and, in addition, exhibit alterations in splicing events when *PRPF8* expression is modulated (Fig. 5e and Supplementary Fig. 2e). Further hierarchical protein‒protein interaction^26^ analysis of these 35 genes (Supplementary Fig. 2e) revealed that fibronectin 1 (FN1) had higher numbers of interactions with other proteins and splicing events, suggesting that FN1, a protein associated with cancer aggressiveness through modulation of the FAK/AKT pathway, is a hub gene in the PRPF8 pathway (Fig. 5f).

### PRPF8 alters FN1 splicing, modulating FAK/AKT pathway activity, cytoskeletal remodeling and the invasion capacity

**Fig. 6:**
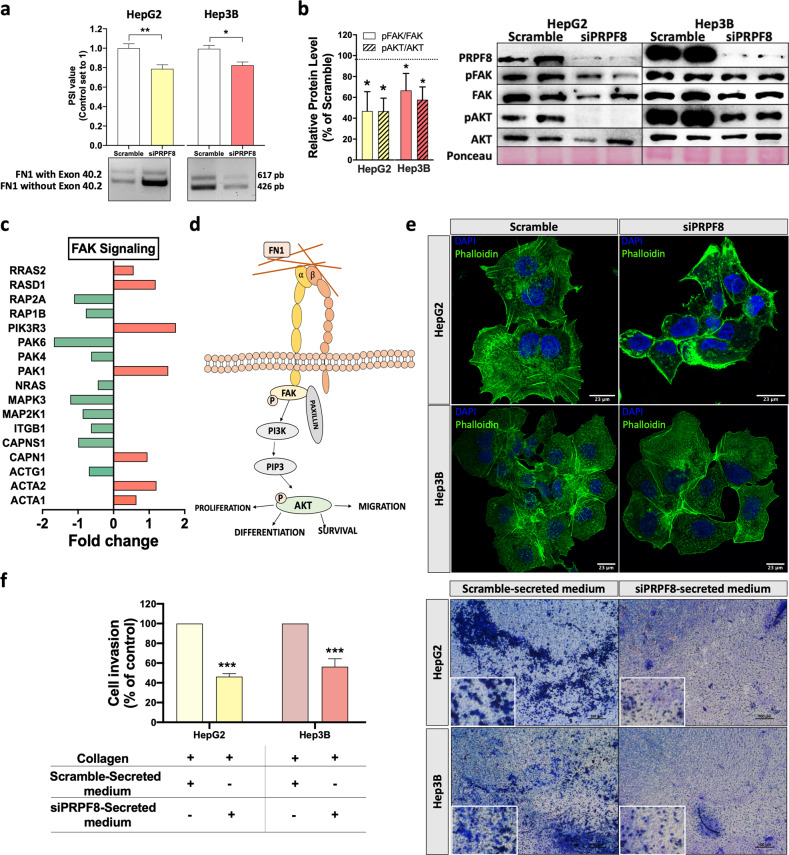
PRPF8 modulates the splicing of FN1, activation of FAK/AKT, remodeling of the cytoskeleton and invasion capacity. **a** In vitro validation of the implication of PRPF8 in exon 40.2 skipping in HepG2 and Hep3B cells, as demonstrated by PCR. PSI (percent spliced in) indicates the inclusion of an exon. **b** Phosphorylation levels of FAK and AKT in response to *PRPF8* silencing in HepG2 and Hep3B cells. The images show representative western blots. **c** Expression of FAK signaling-related genes in response to *PRPF8* silencing. RNAseq data from HepG2 cells were obtained and analyzed. **d** Schematic representation of integrin-mediated FAK/AKT signaling. **e** Confocal fluorescence microscopy analysis of stress fibers (using phalloidin dye) in response to *PRPF8* silencing in HepG2 and Hep3B cells. Nuclei were stained with DAPI (blue). **f** Invasion capacity of HepG2 and Hep3B cells using collagen membranes incubated with conditioned medium from scramble- or *siPRPF8*-treated cells. The data are presented as the mean ± SEM of *n* = 3–5 independent experiments. The asterisks (*p < 0,05; **p < 0,01) indicate statistically significant differences.

RNAseq analysis revealed that *PRPF8* expression is associated with alterations in multiple splicing events in the FN1 gene, especially splicing events associated with exon 40.2 (Fig. 5g). In particular, low levels of *PRPF8* were associated with a lower probability (lower PSI levels) of inclusion of exon 40.2, which encodes important integrin-interacting regions (Fig. 5h). Indeed, the expression levels of FN1 variants containing exon 40.2 were significantly correlated with the expression levels of PRPF8 in the retrospective cohort (Supplementary Fig. 3a). These associations were validated in vitro through silencing and rescue experiments. In particular, the expression of PRPF8 was reintroduced into (rescued in) cells previously treated with PRPF8 siRNAs, in which PRPF8 expression was silenced (Fig. 5i). These results showed that PRPF8 silencing induced a reduction in the PSI value of FN1 exon 40.2 inclusion, while restoration of PRPF8 expression in silenced Hep3B cells by PRPF8 overexpression restored the PSI value of FN1 in parental cells (Fig. 5j). The implication of PRPF8 in FN1 splicing was further corroborated in two different cell lines by demonstrating a significant reduction in the rate of exon 40.2 inclusion in response to *PRPF8* silencing in both the HepG2 and Hep3B cell lines (Fig. 6a), therefore demonstrating the direct implication of PRPF8 in FN1 exon 40.2 inclusion. On the other hand, PRPF8 silencing did not impact total FN1 gene expression, as demonstrated by qPCR analysis of FN1 expression in *PRPF8*-silenced HepG2 and Hep3B cells (Supplementary Fig. 3b). Remarkably, the inclusion of exon 40.2 (as determined by the high PSI value of this event) seems to be a common hallmark of tumor pathologies, including HCC, as demonstrated by analysis of this splicing event in different tumors (tumor vs. normal tissue) in the TCGA SpliceSeq database, in which FN1 exon 40.2 exhibited a higher PSI (a higher percentage of presence) in different types of cancer tissues compared to normal tissues (Supplementary Fig. 3c). In addition, high inclusion of exon 40.2 (a high PSI value) was associated with lower overall survival in HCC patients in the retrospective and TCGA cohorts (Supplementary Fig. 3d). In vitro, *PRPF8* silencing reduced the phosphorylation of FAK and AKT in HepG2 and Hep3B cells 24 h after *siPRPF8* treatment (Fig. 6b) and modulated the expression of several genes involved in FAK signaling, including *ACTA1*, *ACTA2*, and *PAK6*. (RNAseq data, Fig. 6c), consistent with the observation that FN1 is an extracellular matrix protein that interacts with cell membrane integrins (Fig. 6d) to modulate cell migration and invasion through the FAK/AKT pathway^27–29^. Indeed, *PRPF8* silencing also induced reorganization of the cytoskeleton by drastically reducing stress fiber formation (Fig. 6e), which is crucial for cell migration and invasion^30^. To further confirm the role of PRPF8 in cell invasion, we investigated whether PRPF8 silencing can block the secretion of proinvasive molecules, including *FN1* splice variants. To this end, we used conditioned medium from *siPRPF8*- and scramble-treated HepG2 and Hep3B cells to generate conditioned collagen membranes to be tested in invasion assays. Specifically, these conditioned collagen membranes were used to test the invasion capacity of untreated HCC cells. Remarkably, naïve (untreated) HepG2 and Hep3B cells exhibited a significantly higher invasion capacity in membranes treated with conditioned medium from scramble-treated cells than in membranes treated with conditioned medium from *siPRPF8*-treated cells (Fig. 6f), demonstrating that *PRPF8* silencing may block the secretion of proinvasive molecules, as could be the case of FN1 splice variants.

## Discussion

Understanding the molecular complexity underlying the development and progression of HCC is an essential milestone to identify cancer-associated processes or events that may be used to develop novel diagnostic, prognostic and/or therapeutic tools. The splicing process has been shown to be altered in the vast majority of cancer types, thus resulting in splice variants with oncogenic potential^3^. From this perspective, our study presents original data demonstrating that the spliceosomal component PRPF8 was overexpressed at the mRNA and protein levels in different cohorts of patients with HCC, in which its expression was associated with key clinically aggressive parameters related to patient overall survival. Indeed, we demonstrate herein that silencing of *PRPF8* can reduce the tumorigenic capacity of different liver cancer cell lines (HepG2, Hep3B, and SNU-387); reduce proliferation, migration, and the formation of colonies and tumorspheres in vitro; and suppress tumor growth in vivo in a preclinical HCC model. We also revealed that the modulation of PRPF8 leads to alterations in the expression and splicing patterns of numerous genes implicated in different key cancer-related pathways (cell cycle, proliferation, etc.). In particular, this study demonstrates that PRPF8 controls the alternative splicing pattern of the FN1 gene, promoting the generation of splice variants with oncogenic potential, inasmuch as *PRPF8* silencing reduced FAK/AKT signaling, stress fiber formation and the cell invasion capacity.

PRPF8 is a crucial component for the appropriate assembly of the active spliceosome in that it is a component of the ribonucleoprotein (RNP) complex U5 snRNP, contributing to splice site recognition, branch point and U4/U6-U5 tri-snRNP formation and splicing catalysis^11,31^. As has been found for other RNA binding proteins (RBPs), which are increasingly documented to be dysfunctional in cancer genomes^13^, mutations in *PRPF8* have been found to be associated with different pathologies, such as autosomal dominant retinitis pigmentosa^32^ and myeloid malignancies^33^. The recurrent somatic mutation of this factor in multiple cancers^13^ suggests that PRPF8 may play an important role in the development and/or progression of tumor cells. Herein, we corroborated and further expanded the data regarding the elevated expression of PRPF8 in HCC samples compared to nontumor adjacent tissues, which was further validated in larger, publicly available cohorts of patients, such as TCGA (mRNA level) and CPTAC (protein level), and concurs with a recently published study^15^. Remarkably, the expression level of *PRPF8* was tightly linked to those of oncogenic HCC-related splice variants (*CCDC50S* and *KLF6SV1*) and with relevant clinical parameters of aggressiveness (number of nodules, differentiation grade and AFP level) in the retrospective cohort and/or in silico cohort. In addition, high PRPF8 expression was associated with a shorter survival time in all the analyzed cohorts (retrospective, TCGA and^15^). Therefore, this study provides novel evidence demonstrating that PRPF8 is associated with the aggressiveness and poor prognosis of HCC, thereby suggesting that *PRPF8* inhibition could be particularly useful in HCC, a steadily increasing type of cancer for which therapeutic options are scarce^1^.

In this regard, it is worth noting that inhibition of *PRPF8* using specific siRNAs can exert strong antitumor effects on HCC cells. Indeed, in vitro *PRPF8* silencing reduced the proliferation of different liver cancer cell lines through the modulation of important CDKs^34^ such as *CDK2*, reduced the migration capacity, and reduced the colony and tumorsphere formation capacity, all of which are important features related to the control of cancer stem cell viability and cancer progression. These results support the findings of previous studies in HCC^8^ and other cancer types^35^ demonstrating a role of PRPF8 in the control of mitosis and protein homeostasis and further expand previous data revealing the involvement of PRPF8 in other cancer-related processes, such as migration, invasion and stem cell maintenance. In addition, this study is the first to show that in vivo silencing of *PRPF8* using a specific siRNA in growing Hep3B xenografts can significantly reduce tumor progression in this preclinical HCC model. These results compare nicely with previous data from our group demonstrating that genetic or pharmacological blockade of SF3B1, another key component for spliceosome function, can reduce HCC aggressiveness in vitro and in vivo^8^ and provide additional support for the relationship between the dysregulated splicing process and HCC development and progression^6^. Remarkably, while normal cells seem to tolerate a reduction in the activity of PRPF8 or other spliceosome components^8,15^, cancer cells seem to be highly sensitive to a reduction in spliceosome activity, paving the way for targeting the splicing machinery to develop novel strategies for the management and treatment of HCC.

PRPF8 availability has been shown to selectively alter the transcription-coupled splicing of a subset of RNAs with weak 5’ splice sites^11^. To gain further insight into the mechanistic association between PRPF8 and HCC pathophysiology, an exhaustive analysis of RNAseq and CLIPseq data available for *PRPF8*-silenced HepG2 cells was carried out. This analysis showed that *PRPF8* modulation led to dysregulation of the expression and splicing pattern of a vast number of genes, most of which are related to the cell cycle and mitotic process —consistent with previous studies^11^— as well as with cellular growth and cellular stress, which is also consistent with earlier work^13^. In particular, analysis of CLIPseq data showed that PRPF8 physically and preferentially interacts with exonic sequences in a large number of nascent mRNAs, most of which correspond to protein-coding genes. Integrated analysis of RNAseq and CLIPseq data revealed that PRPF8 can physically interact and directly control the splicing pattern of 35 genes, including *FN1*, *HNRNPC*, *ALB* and *RPS27A*, thus suggesting that PRPF8 silencing may reduce HCC aggressiveness by altering the splicing of key cancer-related genes.

One of the PRPF8 target genes with a more drastic alteration in response to *PRPF8* modulation was FN1, an extracellular matrix glycoprotein involved in cell proliferation, embryogenesis, wound healing, host defense, epithelial-mesenchymal transition (EMT) and metastasis, as well as oncogenic transformation^36^. During hepatocarcinogenesis, FN1 has been shown to be upregulated to activate the EMT process, in turn inducing the upregulation of Snail, N‐cadherin, vimentin, matrix metalloproteinase (MMP)2 and phospho‐Smad2, as well as the acquisition of cell migratory behavior. In addition, FN1 increases the expression of vascular endothelial growth factor C-mediated lymphangiogenesis through FAK activation^37^. We show that the presence of PRPF8 is instrumental for the inclusion (in other words, the reduced skipping) of FN1 exon 40.2, a cassette exon shown here to be more prevalent in tumor samples than in normal tissues in a wide variety of cancer types. Exon 40.2 encodes an important region in the FN1 domains that is recognized by cellular integrins^36^ to promote cancer cell motility through FAK/AKT signaling and stress fiber formation^38^. Consistent with this hypothesis, this study demonstrates that *PRPF8* silencing reduces the inclusion of FN1 exon 40.2; inhibits FAK/AKT phosphorylation; alters the expression of several genes involved in FAK signaling; such as *MAP2K1*^39^ and *ITGB1*^40^; and impedes stress fiber formation, all of which are essential for cell migration and invasion^30^, two processes of paramount relevance to HCC. In addition, we demonstrated herein that HCC cells exhibit a significantly lower invasion capacity in membranes treated with conditioned medium from *siPRPF8*-treated cells, demonstrating that PRPF8 silencing may block the secretion of pro-invasive molecules, likely including *FN1* splice variants.

In conclusion, this study reveals new conceptual and functional avenues in HCC, with potential therapeutic implications, by demonstrating a central role of the spliceosome component PRPF8 in hepatocarcinogenesis in which it is overexpressed and associated with aggressive parameters and reduced survival in HCC patients. Indeed, a decrease in *PRPF8* expression is associated with reduced aggressiveness of liver cancer cells in vitro and in vivo and with altered expression and splicing patterns of key cancer-related genes. Finally, PRPF8 is associated with the migration and invasion capacities of HCC cells by modulating FN1 splicing, FAK/AKT signaling and cytoskeletal remodeling, findings that pave the way for targeting this novel PRPF8-related pathway for the development of more specific tools for the management and treatment of HCC patients.

## Supplementary information

Supplemental Material
