# Supplementary material for: PRPF8 increases the aggressiveness of hepatocellular carcinoma by regulating FAK/AKT pathway via fibronectin 1 splicing
Source: Exp Mol Med. 2023 Jan 6;55(1):132–42. doi: 10.1038/s12276-022-00917-7 (PMC9898568; doi:10.1038/s12276-022-00917-7)
Supplement: Supplementary file 1 — Supplemental Material [file 12276_2022_917_MOESM1_ESM.pdf]

RNA isolation and retrotranscription

Total RNA from FFPE tissues was isolated using the Maxwell FFPE Purification Kit (Promega), and total RNA from cell lines was isolated using TRI Reagent (Sigma-Aldrich). RNA extraction, quantification and reverse transcription were performed following previously reported protocols [1-3]. RNA extraction was followed by DNase treatment (Promega, Barcelona, Spain). The amount and purity of RNA recovered were determined using the NanoDrop 2000 spectrophotometer (Thermo Fisher). Finally, RNA (1µg) was reverse transcribed to cDNA using the RevertAid First-Strand cDNA Synthesis Kit (Thermo Fisher).

RNA expression analysis by microfluidic-based qPCR dynamic array and conventional qPCR

RNA expression levels of the transcripts of interest (PRPF8, molecular markers, splicing variants, housekeeping genes) were determined by a microfluidic-based qPCR dynamic array [2-5] in tissue samples and by conventional qPCR in cell lines and xenografted tumors. Specific primers for human transcripts (Supplementary Table 1) were specifically designed with Primer3 software (Applied Biosystems, Foster City, CA). Preamplification, exonuclease treatment, and qPCR dynamic array were implemented following manufacturer's instructions using the Biomark System (Fluidigm, San Francisco, CA). Conventional qPCR reactions were carried out using the Stratagene Mx3000p system with the Brilliant III SYBR Green Master Mix (Stratagene, La Jolla, CA) [1,3]. In the case of tissue samples, the expression level of each transcript was adjusted by a normalization factor (NF) obtained from the expression levels of two housekeeping genes (ACTB and GAPDH) using Genorm 3.3 [6]. In the case of *in vitro* assays and the *in vivo* preclinical model, the expression level of each transcript was adjusted by the expression of ACTB. In all cases, these housekeeping genes exhibited a stable expression among experimental groups.

Semi-quantitative PCR

To determine the presence of FN1 splicing events in liver cancer cell lines, semi-quantitative PCR was implemented by using specific primers designed with Primer3 (Supplementary Table 1) in the T100™ Thermal Cycler (Bio-Rad, Madrid, Spain). Specifically, 50 ng of cDNA were denatured at 95ºC for 3 minutes, followed by 45 cycles of 95ºC for 30 seconds, 57ºC for 30 seconds, 72ºC for 30 seconds, and a final extension at 72ºC for 5 minutes. The PCR products were analysed by electrophoresis in 2% agarose gel, and the results were documented by the Molecular Imager Gel Doc™ XR-Universal H (Bio-Rad, Madrid, Spain).

Western blotting

Liver cancer cell lines were processed to analyze protein levels by western blot after 24 h of PRPF8 siRNA transfection, as previously described [7]. Briefly, 150,000 cells were seeded in 12-well plates and proteins were extracted using pre-warmed Sodium Dodecyl Sulfate-Dithiothreitol (SDS-DTT) buffer (62.5 mM Tris-HCl, 2% SDS, 20% glycerol, 100 mM DTT, and 0.005% bromophenol blue). Then, proteins were sonicated for 10 s and boiled for 5 min at 95 °C. Proteins were separated by SDS-PAGE and transferred to nitrocellulose membranes (Millipore, Billerica, MA, USA). Membranes were blocked with 5% non-fat dry milk in Tris-buffered saline/0.05% Tween-20 and incubated overnight with the specific primary antibodies for PRPF8 (ab79237, Abcam, Cambridge, UK), phospho-FAK (Tyr397) (#700255, Thermofisher), FAK (#39-6500, Thermofisher), phospho-AKT (#4060S, Cell Signaling, Barcelona, Spain), AKT (#9272S; Cell Signaling). Secondary horseradish peroxidase (HRP)-conjugated goat anti-rabbit Immunoglobulin G (# 7074S; Cell-Signaling,) were used. Proteins were detected using an enhanced chemiluminescence detection system (GEHealthcare, Madrid, Spain) with dyed molecular weight markers (Bio-Rad, Madrid, Spain). A densitometry analysis of the bands obtained was carried out with ImageJ software, using total protein levels (Ponceau staining), or total FAK and AKT levels as normalizing factors. Ponceau has been recognized as a better loading control than most of the proteins used as loading controls[8-10]. All experiments were performed, at least, with three independent cell preparations.

Measurements of cell proliferation

Cell proliferation was determined by Alamar Blue-based assays (Thermo Scientific) in all cell lines [1,3,7]. Briefly, 10,000 cells were seeded on each well (96-wells plates) and serum-starved for 24h. Cell proliferation in response to silencing was evaluated every 24h for 3 days. In all experiments, cells were seeded per quadruplicate.

Measurement of cell migration capacity

Cell migration was evaluated by wound healing assay [1,3,7]. Briefly, silenced cells were plated in 24-wells plates in triplicates and, when confluent, serum starved for 1h. A wound was made in the center of each well and the wound-healing capacity determined after 18h. Cells were cultured in serum-free medium to prevent proliferation. In all experiments, cells were seeded in quadruplicate and analysed by ImageJ (FIJI) software.

Clonogenic assay

For clonogenic assays, 2,000 silenced cells were seeded in 6-well plates and incubated for 10 days at 37°C and 5% CO2 [3]. Finally, cells were stained with crystal violet solution (6% glutaraldehyde, 0.5% Violet Crystal) and the number of colonies formed (corresponding to accumulations of more than 50 cells) analyzed using ImageJ. In all cases, the experiments were performed in triplicate.

Tumorspheres formation

To determine the ability of the cell lines to form tumorospheres after the silencing of PRPF8, 10,000 cells were seeded for SNU-387 and 5,000 cells for Hep3B and HepG2, in 24-well Corning Costar Ultra-Low Attachment multiwell plates (Sigma-Aldrich) [1-3]. Cells were seeded in medium with 10% FBS and supplemented with growth factors (FGF, EGF, B27), which were refreshed every 4 days. After 10 days, the number and size of the tumorospheres were analyzed and the mean area of the tumorospheres was determined using Image J. In all cases, the tests were performed in duplicate.

RNAseq data analysis

RNAseq data from PRPF8-shRNA knockdown HepG2 cells was available through ENCODE Portal database [11,12]. Differential expression quantifications (ENCFF902YDX) were analysed by cuffdiff 2.2 software and mapping assembly in hg19 genome. Significantly differentially expressed genes (DEG) were analysed to explore the involvement of these genes in cellular pathways by the use of IPA (QIAGEN Inc., https://www.qiagenbio- informatics.com/products/ingenuity-pathway-analysis) [13]. In addition, differentially splicing quantifications (ENCFF985ELI) were analysed, by rmats sotfware and mapping assembly in hg19 genome, in RNAseq to identify key splicing events when PRPF8 was silenced or not in HepG2 cell line.

eCLIP data analysis

Data from eCLIP experiment on HepG2 against PRPF8 was available through ENCODE Portal database [11,12]. ENCORI (an open-source platform for studying the RBP-mRNA interactions from CLIP-seq data) was used to obtain the list of genes that interact with PRPF8 [14,15]. We used RCAS, an R/Bioconductor package which have been reported in [16], to provide a contextual knowledge of the functional aspects of different biological events that involve RNAs.

References

1 Rincon-Fernandez, D. *et al.* In1-ghrelin splicing variant is associated with reduced disease-free survival of breast cancer patients and increases malignancy of breast cancer cells lines. *Carcinogenesis* **39**, 447-457 (2018).

2 Del Rio-Moreno, M. *et al.* Dysregulation of the Splicing Machinery Is Associated to the Development of Nonalcoholic Fatty Liver Disease. *J. Clin. Endocrinol. Metab.* **104**, 3389-3402 (2019).

3 Lopez-Canovas, J. L. *et al.* Splicing factor SF3B1 is overexpressed and implicated in the aggressiveness and survival of hepatocellular carcinoma. *Cancer Lett.* **496**, 72-83 (2021).

4 Gahete, M. D. *et al.* Changes in Splicing Machinery Components Influence, Precede, and Early Predict the Development of Type 2 Diabetes: From the CORDIOPREV Study. *EBioMedicine* **37**, 356-365 (2018).

5 Jimenez-Vacas, J. M. *et al.* Dysregulation of the splicing machinery is directly associated to aggressiveness of prostate cancer. *EBioMedicine* **51**, 102547 (2020).

6 Vandesompele, J. *et al.* Accurate normalization of real-time quantitative RT-PCR data by geometric averaging of multiple internal control genes. *Genome Biol.* **3**, RESEARCH0034 (2002).

7 Jimenez-Vacas, J. M. *et al.* Spliceosome component SF3B1 as novel prognostic biomarker and therapeutic target for prostate cancer. *Transl. Res.* **212**, 89-103 (2019).

8 Kim, E. *et al.* Promotion of growth factor signaling as a critical function of beta-catenin during HCC progression. *Nat. Commun.* **10**, 1909 (2019).

9 Mahameed, M. *et al.* Pharmacological induction of selective endoplasmic reticulum retention as a strategy for cancer therapy. *Nat. Commun.* **11**, 1304 (2020).

10 Bowles, K. R. *et al.* ELAVL4, splicing, and glutamatergic dysfunction precede neuron loss in MAPT mutation cerebral organoids. *Cell* **184**, 4547-4563 e4517 (2021).

11 Consortium, E. P. An integrated encyclopedia of DNA elements in the human genome. *Nature* **489**, 57-74 (2012).

12 Davis, C. A. *et al.* The Encyclopedia of DNA elements (ENCODE): data portal update. *Nucleic Acids Res.* **46**, D794-D801 (2018).

13 Kramer, A., Green, J., Pollard, J., Jr. & Tugendreich, S. Causal analysis approaches in Ingenuity Pathway Analysis. *Bioinformatics* **30**, 523-530 (2014).

14 Zhou, K. R., Liu, S., Cai, L. & Bin, L. ENCORI: The Encyclopedia of RNA Interactomes (2021).

15 Li, J. H., Liu, S., Zhou, H., Qu, L. H. & Yang, J. H. starBase v2.0: decoding miRNA-ceRNA, miRNA-ncRNA and protein-RNA interaction networks from large-scale CLIP-Seq data. *Nucleic Acids Res.* **42**, D92-97 (2014).

16 Uyar, B. *et al.* RCAS: an RNA centric annotation system for transcriptome-wide regions of interest. *Nucleic Acids Res.* **45**, e91 (2017).

Supplementary Table 1

Supplementary Table 1. Specific primers for human transcripts used in this study. NCBI accession number, primers sequences and expected product sizes are included.

Gene	Accession Number	Primer Sequence (Sense)	Primer Sequence (Antisense)	Product Size (bp)
PRPF8 (Prp8)	NM_006445.3	TGCCCACTACAACCGAGAA	AGGCCCGTCCTTCAGGTA	139
ACTB	NM_001101	ACTCTTCCAGCCTTCCTTCCT	CAGTGATCTCCTTCTGCATCCT	176
GAPDH	NM_002046	AATCCCATCACCATCTTCCA	AAATGAGCCCCAGCCTTC	122
CDK2	NM_001798.4	GCTCTCACTGGCATTCCTCTT	GAGGTTTAAGGTCTCGGTGG	109
CDK4	NM_000075.3	ACAGTTCGTGAGGTGGCTTT	TACCTTGATCTCCCGGTCAG	111
KLF6SV1	NR_027653.2	CGGTGTGCTTTCGGAAGTG	CCTCGCCAGGGAAGGAGAA	104
KLF6	NM_001300.6	GCTGTCTGCTCTGTCCATCA	AGCATTCTGTTCAGGCCACT	80
CCDC50S	NM_174908.4	GCTGGCTATTGAGGCAGAG	TGGCTTCATTCCTCCATCTT	178
CCDC50	NM_174908.4	CGCATTCAGGAGAAGAAGGA	GCAGGGAACTCTGGAAAGTG	101
BCL-XL	NM_138578.3	GATGGCCACTTACCTGAATGA	TGCTGCATTGTTCCCATAGA	94
FN1 (EXON 40.2)	NM_212482.4	TTCCACACCCCAATCTTCAT	CGAGTCATCCGTAGGTTGGT	617/426
FN1 (TOTAL)	NM_212482.4	ACCGTGACAGGAGAGACGA	GAGACCCAGGAGACCACAAA	98

Supplementary Fig. 1

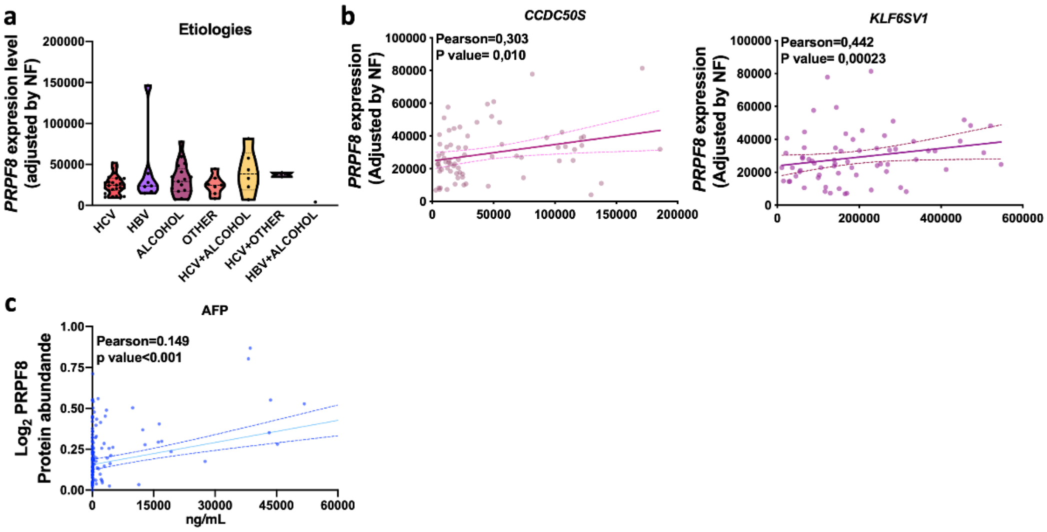

Supplementary Fig. 2

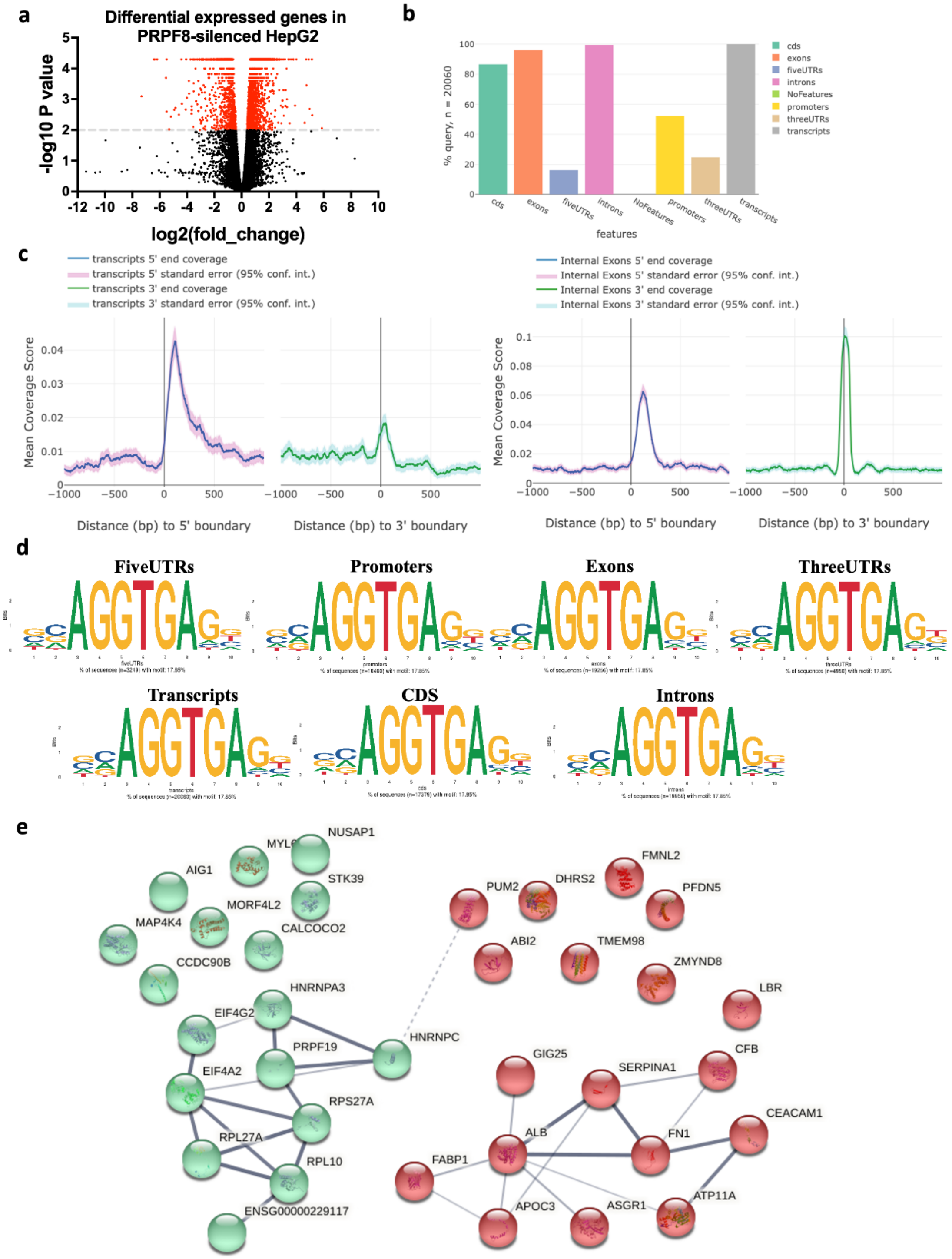

Supplementary Fig. 3

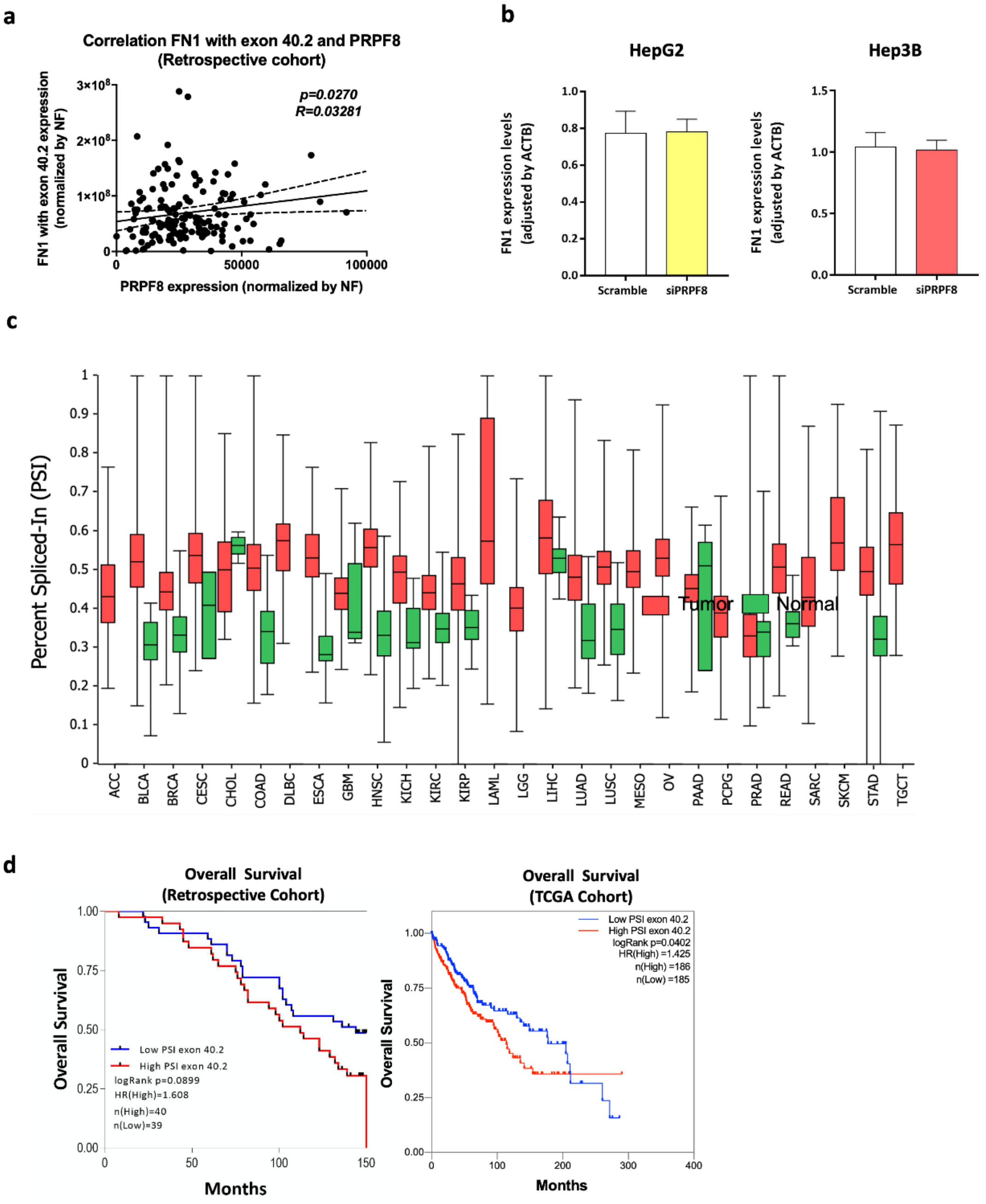

Supplementary Figure legends

Supplementary Fig. 1. Associations and correlations between PRPF8 expression and splicing variants and clinical features of patients with HCC. (a) Expression of *PRPF8* in HCC samples from patients with different etiologies in the retrospective cohort. (b) Correlation between the mRNA expression levels of PRPF8 and oncogenic splicing variants (CCDC50S and KLF6SV1) in HCC samples from the retrospective cohort. (c) Correlation between PRPF8 protein levels and AFP levels in the CPTAC cohort.

Supplementary Fig. 2. RNAseq and CLIPseq revealed that *PRPF8* silencing alters the expression and splicing of multiple genes in HepG2 cells. (a) RNAseq data from *PRPF8*-silenced vs. control HepG2 cells were analyzed to show the volcano plot of altered genes in response to *PRPF8*-silencing. (b) RNA Centric Annotation System (RCAS) in CLIPseq against PRPF8 in HepG2 revealed number of regions that overlap with different kinds of gene features. (c) The coverage profile of regions at/around Transcription Start/End Sites and regions at Exon - Intron Boundaries in CLIPseq against PRPF8 in HepG2. (d) Percentage of sequences with motif in different regions of gene in CLIPseq against PRPF8 in HepG2. (e) Genes directly targeted and regulated by PRPF8. Integration of RNA-Seq and CLIPseq data revealed that PRPF8 can directly bind and regulate the splicing pattern of 35 genes. STRING analysis of these 35 genes suggests two functional clusters directly targeted by PRPF8.

Supplementary Fig. 3. Implication of PRPF8 in FN1 splicing and alteration of FN1 splicing in tumor tissues. (a) Correlation between PRPF8 expresion and FN1 splicing variants harbouring exon 40.2 expression in the retrospective cohort of samples, determined by qPCR. (b) Expression of total FN1 in *siPRPF8*-treated and scramble HepG2 and Hep3B cells, determined by qPCR. (c) Abundance of the skipping of FN1 40.2 exon in tumor vs normal tissues. Data from TCGASpliceSeq (compendium of alternative mRNA splicing in cancer). ACC, Adrenocortical carcinoma; BLCA, Bladder Urothelial Carcinoma; BRCA, Breast invasive carcinoma; CESC, Cervical squamous cell carcinoma and endocervical adenocarcinoma; CHOL, Cholangiocarcinoma; COAD, Colon adenocarcinoma; DLBC; Lymphoid Neoplasm Diffuse Large B-cell Lymphoma; ESCA, Esophageal carcinoma; GBM, Glioblastoma multiforme; HNSC, Head and Neck squamous cell carcinoma; KICH, Kidney Chromophobe; KIRC, Kidney renal clear cell carcinoma; KIRP, Kidney renal papillary cell carcinoma; LAML, Acute Myeloid Leukemia; LGG, Brain Lower Grade Glioma; LIHC, Liver hepatocellular carcinoma; LUAD, Lung adenocarcinoma; LUSC, Lung squamous cell carcinoma; MESO, Mesothelioma; OV, Ovarian serous cystadenocarcinoma; PAAD, Pancreatic adenocarcinoma; PCPG, Pheochromocytoma and Paraganglioma; PRAD, Prostate adenocarcinoma; READ, Rectum adenocarcinoma; SARC, Sarcoma; SKCM, Skin Cutaneous Melanoma; STAD, Stomach adenocarcinoma; TGCT, Testicular Germ Cell Tumors. (d) Overall survival in the retrospective and TCGA cohorts, respectively, categorized as high and low PSI of Exon 40.2 groups based on the median Exon 40.2 PSI, and analyzed by long-rank-p-value method.
